# Treatment of Inflammation of Joints by Permanent Extension

**Published:** 1878-01

**Authors:** Nicholas Senn

**Affiliations:** Milwaukee, Wis.


### THE TREATMENT OF INFLAMMATION OF JOINTS BY PERMANENT EXTENSION.

By Nicholas Senn, M. D., Milwaukee.

[*Read before the Rock River Medical Society of Wisconsin.*]

Among the different methods of applying permanent extension I shall limit myself to that by the weight and pulley as the simplest, most practical and efficient. Like so many of the most important inventions in surgery, it is of comparatively recent introduction, possessing a short but eventful histoty, a history intimately associated with the names of the greatest surgeons of the last century. Sir Benjamin Brodie, whose vast experience, close observation and originality of thought so rapidly developed the resources of surgical appliances, first employed extension in the treatment of joint affections. Observing the frequency of spontaneous dislocation of the head of the femur after coxitis, it occurred to him that this formidable result might be avoided, if extension could be applied during the progress of the disease, and that thus spasmodic muscular contractions might be relieved. He applied extension by fastening a belt with buckle around the thigh above the condyles of the femur ; to this belt the extension cord was tied which passed, with the weight, over a simple pulley at the foot of the bed. The necessary counter-extension was made by encircling the pelvis with a broad leather belt and fastening it to the opposite side of the bed with cords. Rude and imperfect as this method of application must have been, it yielded in his hands the desired results. Experience soon demonstrated that it not only prevented dislocation, for which it was originally intended, but it also removed pain, and modified the process of inflammation. Through his influence this new treatment was soon adopted by his contemporaries ; but, on account of want of skill or the imperfection of the appliance, as taught by Brodie himself, it soon fell into disrepute. In 1854, it was presented as something entirely new by Gustav Ross, in a short article, published in the *Deutsche Klinik*, No. 9, 1854, entitled, " Ueber ein neues Behandlungsprincip der Gelenkentzündung." This time it was proposed as a substitute for the *brisement forcé*, as taught by Bonnet, for correcting deformities. *brisement forcé*, or rapid and forcible extension, was at that time advised during the progress of the inflammatory process. This heroic treatment could not fail to frequently produce disastrous results, which induced Ross to substitute for it the more rational means of gradual and permanent extension. He strongly urged its advantages in being applicable during the early stages of disease, and its influence in abating in place of aggravating the co-existing inflammation. In America, it found ready introduction through men like Wallace, Davis, Taylor, Pancoast, and Sayre. In Europe it has come into general use through the researches and untiring labors of Barwell, Volkmann, Langenbeck, and Hueter. There is at present, perhaps, no other surgical appliance in more general use over the entire world than permanent extension for the treatment of joint affections. While most surgeons resort to it both in hospital and private practice, there are a few exceptions, prominent among them Dr. Louis Bauer, formerly of New York, now of St. Louis. He believes that the only beneficial effect, if any, consists in fixation; that it acts as an additional irritant to the already irritated and contracted muscles, aggravating inflammation.

The accumulated experience of many reliable observers, however, has conclusively demonstrated that gradual extension—

(1) removes pain; (2) corrects deformity; (3) directly influences the course and result of inflammation.

Extension by weight and pulley has generally been applied to affections of the knee and hip joints, although it has been used with equal success in other parts of the body. Volkmann has used it extensively in Potts' disease of the spine. In this disease we have all witnessed the prompt disappearance of the excruciating pain after ex tension by some mechanical means sufficient to separate the inflamed surfaces of the bodies of the affected vertebræ. I have now under my care, at the Milwaukee Hospital, a little girl, suffering from a chronic inflammation of the shoulder joint, accompanied by a rotation of the humerus outward, who is rapidly improving after a few weeks of extension.

The mode of application is the following: The skin should be shaved before applying the adhesive plaster, which should be fresh and spread on Canton flannel. Two strips, each sufficient in width to cover one-third of the circumference of the limb, should be applied to each side of the limb and extend from the inflamed joint to below the extremity of the limb, where it is fastened over a block of wood of sufficient length to prevent injurious pressure over the bony prominences of the joint. To the center of this block of wood a short cord is fastened, which runs over a pulley at a point corresponding to the axis of the limb, with the necessary weight attached. I am always in the habit of cutting the upper portions of the extension straps into several strips to secure a better hold, and to bandage the limb from the distal end to the joint affected, with a roller over which is applied flour paste to prevent it from slipping. The plaster should be applied evenly and smoothly, and the bony prominences, if necessary, protected by cotton. The pulleys should be large, with a deep groove. It often is convenient to use more than one pulley—one near the mattress, and the other on the top of the foot of the bed, or in the ceiling. The weight must vary according to the age of the patient, and the amount of muscular contraction to be overcome. It will vary from three to twenty pounds. The best substances to regulate the amount most accurately are sand and shot. As a rule, it is best to begin with a small weight and gradually increase it, until the desired effect, muscular relaxation, has been accomplished. When muscular action has ceased, it is well to diminish the weight. An important fact to remember is this: Always begin extension in the direction of the deformity. I am satisfied by personal observation, that this rule is not generally observed. Extension should, at once, diminish pain instead of increasing it, as will inevitably be the case if this rule be neglected. The very object of extension is to tire out the contracted muscles gradually, and not suddenly. If extension increase the suffering, it has not been properly applied, or the case is not one appropriate for its use. In acute affections of joints, contraction is one of the earliest symptoms. In acute coxitis we may find, at our first visit, the thigh contracted at a right angle to the pelvis. In such cases, the extension must be modified to meet the indications in each individual case. I generally proceed as follows: I have a double-inclined plane made, extending from the foot to the tuberosity of the ischium, the angle corresponding to that of the limb. The two planes are connected by hinges; the base consists of a board with notches, into which the lower plane fits, for the purpose of increasing or diminishing the angle of the planes. Two broad strips of adhesive plaster are now applied to the sides of the thigh, extending from the hip-joint to below the knee, where they are fastened over a block of wood, to which is applied the extension cord. The pulley is fastened into the ceiling at a point which corresponds to the axis of the femur. By bringing the lower plane into the lower notches, the angle can be gradually diminished until the limb is in a straight position—when extension in the usual manner can be applied. The double-inclined plane is not only convenient for the purpose of straightening the limb, but acts as a splint fulfilling another indication in the treatment of all inflamed joints, second in importance only to extension, viz., fixation. The inclined plane can be used with the same benefit and for the same purposes, if the extension is to be applied to the leg, in cases of inflammation of the knee-joint. Fixation and extension should always be combined. Extension alone is insufficient to insure the desired immobility of the joint, hence it is almost always necessary to call into requisition some additional means to accomplish this object. Besides the inclined planes, I have found leather splints of great value. In cases of hip disease, after the limb is brought nearly to a natural position, I take a piece of thick sole leather extending from the axilla to three inches below the foot. It must be sufficiently wide to cover one-half of the circumference of the body, thigh and leg. After it is thoroughly soaked in water, the inner surface is covered with one or two layers of cotton-batting, and the splint applied to the outer side of the limb. Care must be taken to mold it accurately to all of the irregularities of the surface. It is retained *in situ* by two or more roller bandages extending from foot to axilla. The bandages are thoroughly impregnated with rye-flour paste. During the time the splint is applied, the patient must be held in a horizontal position over a table, by several assistants, extension being made at the same time. After the splint is applied, the patient is placed in bed, and extension by weight and pulley continued until the application becomes dry, which usually takes from one to two days. By cutting the bandages on the inner side of the leg and side of the body, introducing eyelets near the cut margins, the splint can be removed and accurately re-applied by lacing. This splint secures perfect fixation, and I prefer it to the more expensive wire-splint of Bauer. In cases of disease of the knee joint, extension can be combined with fixation by means of a posterior splint, with foot-board and movable joint at the knee. For extension at the ankle joint, a plaster-of-Paris shoe must be applied, into which the extension straps are incorporated. The equal pressure from this dressing produces results which surpass those when adhesive plaster is relied upon.

It is interesting to observe that while extension has been used with the best results throughout the whole civilized world, authorities differ in regard to its mode of action. Brodie used it to prevent dislocation; Ross to prevent deformity; to-day we attribute to it, in addition, direct antiphlogistic properties. Prof. Volkmann believes that the articular surfaces are separated by elongation of the ligaments, and so firm are his convictions in this respect that he terms this treatment "Distractionsmethode." Bardeleben, and most American surgeons, sustain his views. Prof. C. Hueter, on the other hand, asserts that its curative influence consists in an increase of intra-articular pressure and immobility. In regard to its effect on intra-articular pressure, Reyher's experiments on cadavers are conclusive. He found by injecting different joints that their greatest capacity was reached in the normal semiflexed physiological position. The capacity for fluid diminished by a deviation from this position. We find, in practice, that patients suffering from inflammation of joints uniformly assume this position. To test the effect of extension in affecting joint capacity, the same experimenter made an opening into the knee joint through the patella, inserting a glass tube, and filling the joint with water. He found the column of water in the tube lowest when the limb was semiflexed. Applying extension in the ordinary manner, the fluid in the tube rose; this effect he attributed to the compression of the joint by the traction of the skin over it. After division of the skin above the joint by a circular cut, the column of water again diminished. Hueter repeated these experiments and corroborates their correctness. Experience at the bedside also confirms these statements. We frequently meet with cases of acute inflammation, attended by copious and rapid effusion, aggravated by extension, while the more chronic cases with relaxation of the structures of the joints are promptly benefitted by it. If it is thought desirable to make extension and avoid this increase of articular pressure, it is important to apply the plaster as far away from the affected joint as possible, and to include the bony prominences of the next distal joint.

When we reflect upon the unyielding character of the ligaments of the large joints, it becomes difficult to conceive how the articular surfaces can be parted by any justifiable amount of extension. This result undoubtedly can be obtained after the disappearance of a copious effusion with relaxation of the ligaments, or after the prolonged use of a heavy weight. It is doubtful whether such separation is ever necessary. The principal use of the weight consists in neutralizing the reflex muscular contractions of the flexor muscles, and in doing this it does not diminish intra-articular pressure, but the pressure between the articular surfaces. That this increased pressure produced by muscular contraction acts unfavorably upon the local process is shown by numerous specimens. The greatest distraction of tissue is uniformly found where the greatest amount of pressure had been exerted. It is also a well-known fact that in neglected cases of inflammation of joints that have existed for an indefinite time, recovery frequently takes place after removal of this pressure by spontaneous dislocation. Extension not only diminishes pressure directly, but, at the same time, by gradual straightening of the limb, changes the point of pressure. I apprehend that this constitutes an important factor in its mode of action.

The empirical use of extension has not only led to frequent disappointment, but has often been the cause of much unnecessary suffering. As with so many other valuable agents, it has been abused by its too indiscriminate employment. A rational use of it must be limited to certain well defined pathological conditions. Volkmann and Langenbeck have used it with great success in the acute inflammation of joints following gunshot injuries. If these or any other traumatic injuries lead to disorganization of the cartilage or ends of the bones, its employment becomes of paramount importance for the safety of the joint. If, on the other hand, the disease is limited to the synovial membrane and ligaments of the joints, and the surgeon is called before decided contraction has taken place, fixation is preferable to extension. In catarrhal inflammation of joints it is useful as an orthopedic remedy, promoting, at the same time, absorption by moderate compression. The best results by extension have been obtained in cases where the disease involved the articular cartilages or the bone itself. These cases are always accompanied by painful muscular contraction, which promptly subsides after its use. Its power to remove pain was recognized by Brodie. Howard Marsh found in children suffering from hip disease, that after he removed the weight during sleep, they would soon become restless and awake with pain. The same effect is also observed if the weight be removed too soon, before all pain and tenderness have subsided. Great care, therefore, must be exercised not to remove the weight too early. I recollect two cases of hip disease where extension was continually applied for over six months. In one case suppuration took place; the abscess appeared on the anterior surface of the thigh below the joint; and fluctuation, which had been apparent for several months, gradually disappeared, and the limb became anchylosed in a good position. In the second case the weight was removed on two different occasions, but as the pain reappeared in a few days, it had to be re-applied. After eight months of treatment, recovery took place, with a good movable joint. In some cases of a strumous diathesis it may become necessary to substitute for the weight the portable apparatus for extension, as Davis', Taylor's, Andrew's, or Sayre's splint. After the acute symptoms have subsided, and the limb is in proper position, one of these splints may be applied during the day, and the patient permitted to be carried into the fresh air, and at night the extension by weight continued.

Extension by splints of any kind is insufficient in recent cases, and can never take the place of the weight and pulley except as an auxiliary measure to complete the treatment.

We must, lastly, consider extension as an orthopedic agent. Employed for this purpose it is a rival to *brisement forcé.* The latter can judiciously be employed only after all inflammatory symptoms have been removed ; extension, on the other hand, is one of the most valuable remedies to subdue inflammation, and consequently is applicable during the early stages of the disease. It not only corrects, but, what is still more important, prevents deformity. *Brisement forcé* can correct angular deformity only ; it is perfectly useless in removing abduction or rotation. Extension, by gradually neutralizing muscular contraction and elongating ligaments, is not only a safer means for restoring the normal angle, but accomplishes at the same time the latter. In old contracted joints, the ligaments on the flexed side have frequently undergone such an amount of thickening and shortening that nothing short of rupture would permit a restoration to the normal position. If immediate and rapid extension be used, aside from the injury inflicted by such heroic treatment, a relapse is frequently inevitable from contraction of the cicatrix, resulting from healing of this subcutaneous wound. Gradual extension, however, causes no new injuries, but simply elongates the contracted tissues. For the application of extension to contractures of the muscles of the hip joint, I would refer to an article on this subject in my report to the State Medical Society, at its last session. In some obstinate cases of fibrous anchylosis it may be well to combine forcible with gradual extension.

There is one condition of the knee joint which positively contra-indicates the use of extension. I refer to partial dislocation of the tibia backwards, the result of a preceding gonitis. Whenever the head of the tibia has been displaced backwards, so that the anterior edge has passed the median line of the articular surface of the femur, the tibia is converted into a lever of the second kind, and the displacement is aggravated by extension.

I conclude by presenting the following propositions for consideration :

I. The weight and pulley constitute the best method for making permanent extension.

II. Extension should always be first made in the line of deformity.

III. Extension operates as an antiphlogistic and orthopædic agent, by removing muscular aud ligamentous contractions.

IV. Permanent extension is most useful in cases of joint affection where the cartilages or the ends of the bones are diseased.

V. As an orthopædic measure, extension should always supersede *brisement forcé*, so long as pain and tenderness exist; in exceptional cases of contracture, it may be profitably combined with forcible extension.

VI. In the treatment of inflamed joints, extension must be combined with fixation.

MILWAUKEE, WIS., Sept. 14th, 1877.

